# Effects of rTMS and tDCS on neuropathic pain after brachial plexus injury: a randomized placebo-controlled pilot study

**DOI:** 10.1038/s41598-022-05254-3

**Published:** 2022-01-27

**Authors:** Erickson Duarte Bonifácio de Assis, Wanessa Kallyne Nascimento Martins, Carolina Dias de Carvalho, Clarice Martins Ferreira, Ruth Gomes, Evelyn Thais de Almeida Rodrigues, Ussânio Mororó Meira, Ledycnarf Januário de Holanda, Ana Raquel Lindquist, Edgard Morya, Cristina Katya Torres Teixeira Mendes, Thaís Castro Gomes de Assis, Eliane Araújo de Oliveira, Suellen Marinho Andrade

**Affiliations:** 1 Aging and Neuroscience Laboratory, Federal University of Paraíba, João Pessoa, Brazil; 2 State Hospital for Emergency and Trauma Senator Humberto Lucena, João Pessoa, Paraíba Brazil; 3 Laboratory of Intervention and Analysis of Movement, Department of Physical Therapy, Federal University of Rio Grande do Norte, Natal, Brazil; 4 Edmond and Lily Safra International Institute of Neuroscience, Santos Dumont Institute, Macaíba, Rio Grande do Norte Brazil

**Keywords:** Neuroscience, Health care, Neuropathic pain

## Abstract

Neuropathic pain after brachial plexus injury (NPBPI) is a highly disabling clinical condition and is increasingly prevalent due to increased motorcycle accidents. Currently, no randomized controlled trials have evaluated the effectiveness of non-invasive brain stimulation techniques such as repetitive transcranial magnetic stimulation (rTMS) and transcranial direct-current stimulation (tDCS) in patients suffering from NPBPI. In this study, we directly compare the efficacy of 10-Hz rTMS and anodal 2 mA tDCS techniques applied over the motor cortex (5 daily consecutive sessions) in 20 patients with NPBPI, allocated into 2 parallel groups (active or sham). The order of the sessions was randomised for each of these treatment groups according to a crossover design and separated by a 30-day interval. Scores for “continuous” and “paroxysmal” pain (primary outcome) were tabulated after the last stimulation day and 30 days after. Secondary outcomes included the improvement in multidimensional aspects of pain, anxiety state and quality of life from a qualitative and quantitative approach. Active rTMS and tDCS were both superior to sham in reducing continuous (*p* < 0.001) and paroxysmal (*p* = 0.002; *p* = 0.02) pain as well as in multidimensional aspects of pain (*p* = 0.001; *p* = 0.002) and anxiety state (*p* =  < 0.001; *p* = 0.005). Our results suggest rTMS and tDCS are able to treat NPBPI with little distinction in pain and anxiety state, which may promote the use of tDCS in brachial plexus injury pain management, as it constitutes an easier and more available technique.

*Clinical Trial Registration*: http://www.ensaiosclinicos.gov.br/, RBR-5xnjbc – Sep 3, 2018.

## Introduction

Traumatic brachial plexus injuries are diagnosed in more than 1% of patients treated in emergency units^1,2^. Neuropathic pain after brachial plexus injury (NPBPI) affects 30 to 90% of patients^3–7^, and occurs due to deafferentation, i.e. a loss of sensory afferent input, most commonly in preganglionic lesions when there is brachial plexus avulsion or in complete lesions^8,9^. The pain is usually severe, mainly located in the forearm and hand, in the form of a continuous burning sensation associated with acute pain paroxysms^10,11^. Since chronic pain results from inadequate plastic changes in the central and peripheral nervous system^12^, non-invasive brain stimulation techniques such as repetitive transcranial magnetic stimulation (rTMS) and transcranial direct-current stimulation (tDCS) have been reported as a therapeutic option^12–14^.

Recent neurophysiological support and previous neuroimaging studies for this possibility comes from reports that rTMS and tDCS over functionally connected regions of the distributed motor network leads to greater enhancements in pain relief, especially given the evidence to modulate the activity of an extensive neuronal network^13^, which includes thalamic nuclei, the limbic system, brain stem nuclei, and spinal medulla^15–18^. However, currently, a direct comparison approach about rTMS and tDCS techniques has not been explored in relation to neuropathic pain (NP) located in the upper limb^12,14,18^.

In the current crossover study, we focused on comparing changes in pain intensity and multidimensional aspects of patients with NPBPI, from sensory, affective and evaluative components of pain aspects using a qualitative and quantitative approach. In light of previous experiments demonstrating changes in cortical activity following rTMS under different painful conditions^12,19,20^, we hypothesised that rTMS over the primary motor cortex would modulate motor network excitability more effectively than tDCS in NPBPI individuals, and produce greater clinical changes, due to the additional motor pathways emerging from rTMS. Lastly, as a secondary analysis, we explored the effects of stimulation over the anxiety and quality of life in this population, which constitute two important nodes within the management of chronic pain^21–23^.

## Methods

### Participants

Participants were recruited from the outpatient clinic of the State Hospital for Emergency and Trauma Senador Humberto Lucena between September and December 2018. Patients aged between 18 and 60 years who scored 4 to 10 points on the Visual Analogue Scale (VAS)^24,25^, considering the last 24 h, with NPBPI refractory to clinical treatment, persistent for at least 6 months, and who received adequate pharmacological treatment for pain with the combination of antidepressants, gabapentinoid antiepileptics and analgesic opioids^26^, for at least 1 month before the study were considered eligible^13^. We applied the DN4 Questionnaire to confirm the presence of NP^27,28^. No changes in medication regimens were allowed during the study. Exclusion criteria were the presence of other neurological or psychiatric diseases, including ongoing major depression, history of substance abuse, in addition to formal contraindications for rTMS and/or tDCS^12,14^. We applied the Beck Depression Inventory (BDI)^29^ to identify and grade depressive symptoms. The selected participants underwent rTMS and tDCS sessions conducted in an institutional neuromodulation laboratory.

### Ethical aspects

This study was carried out respecting the ethical principles expressed in the Declaration of Helsinki^30^, and all participants voluntarily signed an informed consent form. The protocol was previously approved by the Research and Ethical Committee of the Health Sciences Center at Federal University of Paraíba (statement 2.563.783), and was registered at ClinicalTrials (ensaiosclinicos.gov.br) with the ID number RBR-5xnjbc (09/03/2018).

### Study design

We conducted a pilot, placebo-controlled, double-blind, randomized, crossover clinical trial in accordance with the CONSORT guidelines^5,31^. Participants were randomly allocated to one of 2 parallel groups: active or sham stimulation, a ratio of 1:1. We used a random number generator in an online randomization program (www.random.org). The allocation was hidden using sequential numbered, opaque and sealed envelopes.

The procedures related to allocation, randomization, evaluation, intervention, and data analysis were carried out by independent researchers who were unaware of each other’s work. Blinding was also extended to patients, who were not aware of the allocation group and hypotheses of the study.

**Figure 1:**
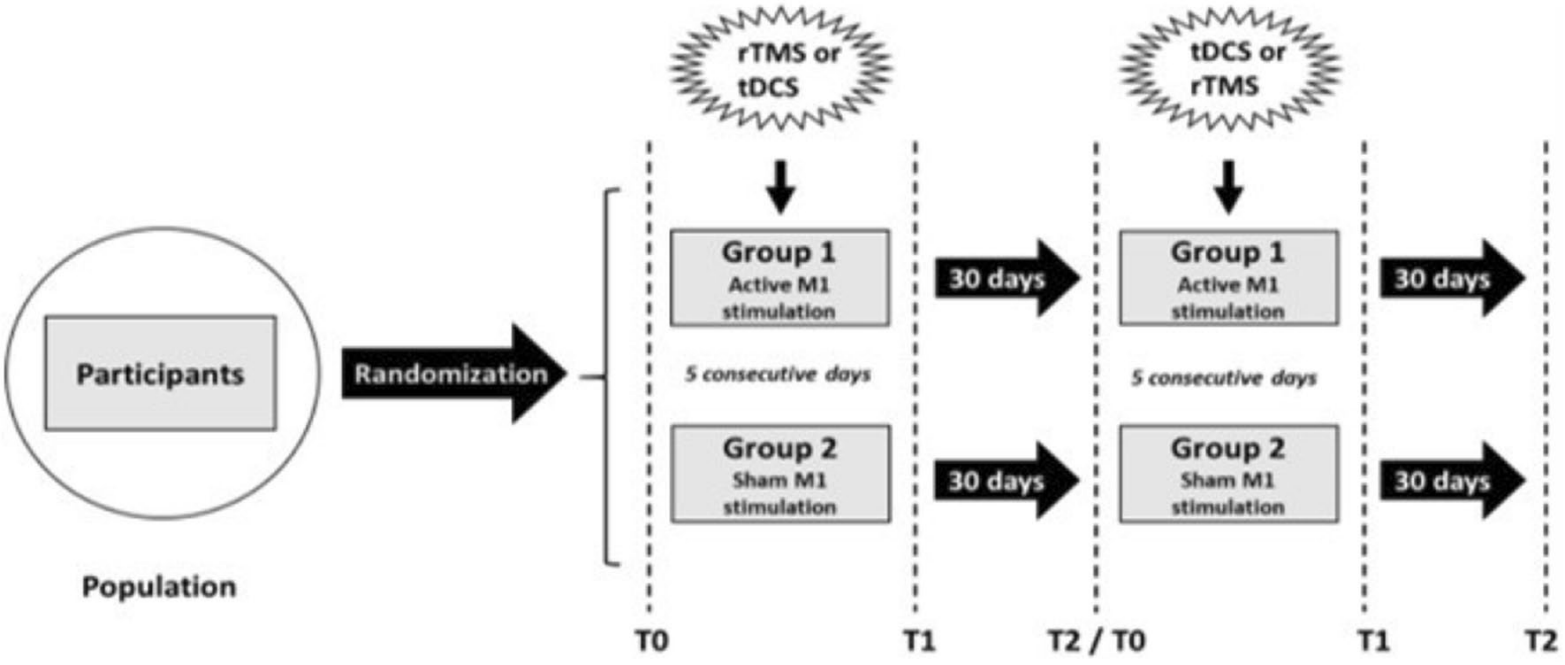
Study design. The treatment protocol included 2 stimulation blocks separated by a 30-day interval. Each block consisted of 5 sessions for 5 consecutive days, during which each patient received repetitive transcranial magnetic stimulation (rTMS) or transcranial direct-current stimulation (tDCS) for 30 min. Each patient underwent 10 stimulation sessions in all. Assessments took place immediately before the first stimulation (T0), after the 5th consecutive stimulation session (T1) and 30 days after (T2). M1 primary motor cortex.

The session order (rTMS and tDCS) was random for each treatment group (active or sham) according to a crossover design. Group 1 received active rTMS followed by active tDCS or active tDCS followed by active rTMS, while group 2 received sham rTMS followed by sham tDCS or sham tDCS followed by sham rTMS (Fig. 1). This design was based on a previous clinical study^13^, and avoided the need for patients to receive placebo and active stimulation in the same crossed arm. The treatment protocol included 2 stimulation blocks separated by a 30-day interval, a period of washout considered sufficient to reduce possible carry over effects and compatible with that reported in a previous study^13,32,33^. Each block consisted of 5 sessions for 5 consecutive days, during which each patient received rTMS or tDCS for 30 min. At the end, each patient received a total of 10 stimulation sessions (2 series of active rTMS/tDCS or 2 series of sham rTMS/tDCS; Fig. 1).

### Assessments and outcomes

In the baseline visit, we performed the structured questionnaire including demographic data, affected side, injury characteristics, DN4 and BDI scores, and current clinical treatment. Primary and secondary outcomes were developed in accordance with IMMPACT recommendations for clinical trials related to chronic pain treatment^23^.

The primary outcome was the improvement in pain intensity measured by the VAS^34,35^. Scores for “continuous pain” and “paroxysmal pain”^10,36^ for the last 24 hours were obtained, providing a score from 0 to 10^12,14,19,35^. Assessments were performed in each stimulation block, immediately before the first stimulation session (T0), after the 5th consecutive stimulation session (T1) and after a 30-day interval (T2).

Secondary outcomes were concurrently assessed with the primary outcome as follows: (1) multidimensional aspect of pain based on Brazilian-Portuguese version of the McGill Pain Questionnaire (MPQ)^37,38^; (2) the anxiety state measured by the State-Trait Anxiety Inventory (state subscale) (STAI-S)^39^; and (3) changes in quality of life assessed by the SF-36 Quality of Life Questionnaire^40^. The safety of rTMS and tDCS was assessed by monitoring the occurrence of adverse effects during treatment, with the application of a checklist at the end of each session.

## Interventions

### Repetitive transcranial magnetic stimulation

The TMS device was a Neuro-MS/D magnetic stimulator (Neurosoft Ltd., Ivanovo, Russia), using an angled and cooled figure-of-8 coil (F8) over M1 contralateral to the painful side. The rTMS parameters were similar to those reported in previous studies^12,18^: 90% RMT, 10 Hz, 2500 pulses per session (25 trains of 10 seconds each, with an interval of 17 seconds) on 5 consecutive days.

The ideal coil position was marked on an elastic cap worn in each session, to signal the target area in M1. This marking was based on: (1) the International 10/20 System; (2) surface anatomy references; and (3) resting motor threshold (RMT) record. The coil was held in position by an articulated support, tangentially oriented to the interhemispheric fissure, and all subjects clearly heard the coil noise.

The M1 hot spot was defined according to the motor function grade in the affected limb. We applied a single TMS pulse in patients with useful hand motor function to elicit a minimal visible contraction in a muscle of the fingers (i.e. abductor pollicis or first dorsal interosseous) in at least 5 of 10 pulses^41,42^. We used hemiface muscles ipsilateral to the painful limb as reference in patients without useful motor function due to the somatotopic cortical proximity in relation to the motor area of the hand^19,43,44^.

The procedures for locating M1 and determining RMT were the same for the rTMS applied in the placebo group. The same F8 coil, but elevated and tilted out of the head, was used to reproduce some of the subjective sensation of rTMS to simulate rTMS sessions, and still avoid current induction in the brain^17,34,41–46^. Since none of the patients had previously experienced rTMS, they had no idea what an active stimulus would feel like^17,34,41–46^.

### Transcranial direct-current stimulation

A battery-powered tDCS stimulator (TCT Research Ltd., Kowloon, Hong Kong) was used. The stimulation protocol was guided by previous studies^14,47^, with the active current applied in M1 contralateral to the painful side. The anode was positioned over C3 or C4 according to (1) the International 10/20 System and (2) surface anatomy references, and the cathode positioned over supraorbital region contralateral to the anode^48^. The electrodes were wrapped by 5 × 7 cm sponges, moistened with saline (NaCl 0.9%), with an applied current of 2 mA, and the current density equivalent to 0.057 mA/cm^2^.

The protocol for tDCS placebo was identical, but the device stopped emitting a current 30 seconds after starting the stimulation. Thus, the effects of active stimulation (slight tingling and itching sensation) were simulated, constituting a reliable blinding method with the effects disappearing soon after the stimulation started^47^.

### Statistical analysis

The analyses were based on the intent-to-treat principle. Clinical and demographic variables were compared at baseline through one-way analysis of variance (ANOVA) for continuous data, or the Chi-squared test for categorical data.

All efficacy measures, corresponding to the primary outcome (mean score of continuous and paroxysmal pain intensity) and all secondary efficacy variables (MPQ scores, STAI-S and SF-36), were analysed by a split plot ANOVA with a mixed effects model. The model included the following explanatory variables: group (rTMS, tDCS or sham stimulation), time (T0, T1 and T2), order of the sessions (rTMS followed by tDCS or tDCS followed by rTMS), and the interaction effect between group and time. The baseline-observation-carried-forward (BOCF) approach was used to handle missing data.

Comparisons between groups were considered post hoc and corrected by the Bonferroni procedure. Fisher’s exact test was used to compare proportions. *p*-values < 0.05 were considered significant in all cases. The effect size was obtained through partial eta-squared and Cohen’s d according to each type of comparison. All analyses were performed by an independent researcher with the IBM SPSS Statistics 22.0 computer program (IBM Corp., Armonk, NY).

## Results

### Patients

**Figure 2:**
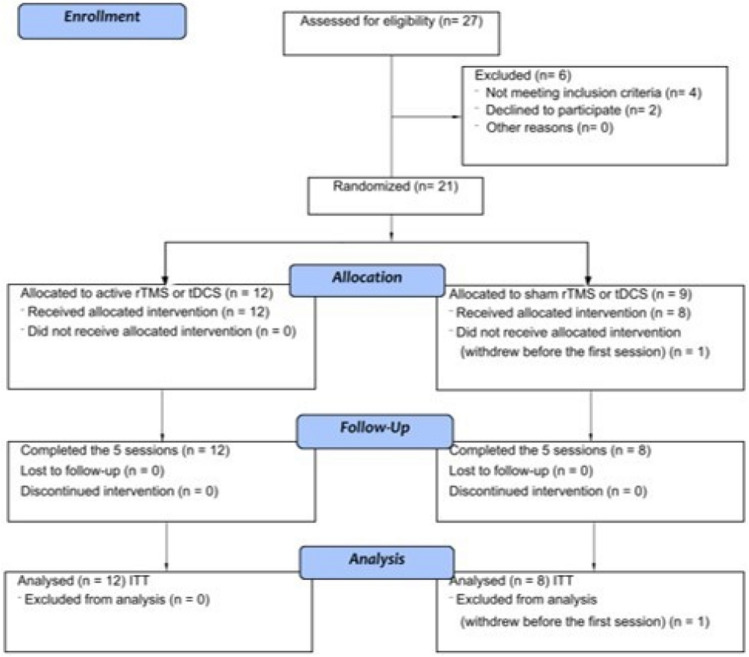
Patient disposition and CONSORT flowchart. ITT intent-to-treat.

We screened 27 patients with NPBPI and refractory treatment, all of them male; 6 patients were excluded for not meeting the eligibility criteria or refusal (Fig. 2). The remaining 21 patients were randomly allocated to groups of active stimulation (n = 9), but one patient withdrew from the trial before the first session was conducted. Data were obtained for 20 patients who underwent at least one active or sham stimulation session (Fig. 2).

Table 1 presents the baseline demographic and clinical characteristics of the participants such as affected side, injury characteristics, DN4 questionnaire, BDI scores, current clinical treatment, pain intensity scores (VAS), MPQ, STAI-S and SF-36. These characteristics were not significantly different between the groups at baseline (T0) (*p* > 0.05) (Table 1).

**Table 1:** Demographic and clinical data at baseline (intent-to-treat population).

	Group 1 (n = 12) (active)	Group 2 (n = 8) (sham)	*p* value
Age (mean ± SD)	34 ± 10	31 ± 7	.466
Gender, % (n)			
Male	100 (12)	100 (8)	
Injury time in months (mean ± SD)	34.01 ± 31.04	44.44 ± 36.86	.468
**Affected upper limb, % (n)**			
Right	58.33 (7)	41.66 (5)	.319
Left	37.50 (3)	62.50 (5)	
**Pain site, % (n)**			
Hand	58.33 (7)	37.50 (3)	.750
Hand and forearm	16.67 (2)	37.50 (3)	
Arm	8.33 (1)	12.50 (1)	
Shoulder	8.33 (1)		
Whole upper limb	8.33 (1)	12.50 (1)	
**Number of affected dermatomes (C5-T1) (mean ± SD)**			
Total affected	4.53 ± 0.87	4.63 ± 0.74	.293
Hypoesthesia	2.35 ± 1.54	2.25 ± 1.58	.534
Anesthesia	2.18 ± 1.70	2.25 ± 1.39	.613
**Affected myotomes (C5-T1), % (n)**			
C5	83.33 (10)	87.50 (7)	.993
C6	100 (12)	100 (8)	
C7	100 (12)	100 (8)	
C8	91.67 (11)	100 (8)	
T1	75.00 (9)	100 (8)	
Horner’s syndrome, % (n)	75.00 (9)	75.00 (6)	.936
**Presence of motor function, % (n)**			
Useful (MRC grade 3–4)	41.66 (5)	37.50 (3)	.850
Residual (MRC grade 1–2)	50.00 (6)	50.00 (4)	
Absent (MRC grade 0)	8.33 (1)	12.50 (1)	
**Location with motor function, % (n)**			
Shoulder	50.00 (6)	50.00 (4)	.787
Elbow	8.33 (1)	25.00 (2)	
Wrist	16.67 (2)	25.00 (2)	
Hand	25.00 (3)	25.00 (2)	
Whole upper limb	8.33 (1)		
**Medications used, % (n)**			
Opioids	100 (12)	100 (8)	.568
Antiepileptics	83.33 (10)	75.00 (6)	
Antidepressants	75.00 (9)	75.00 (6)	
**Physiotherapy, % (n)**			
Yes, at the moment	66.67 (8)	87.5 (7)	.388
No, but already did	16.67 (2)	12.5 (1)	
Never did	16.67 (2)		
BDI (mean ± SD)	17.29 ± 8.62	20.88 ± 7.18	.319
DN4 (mean ± SD)	7.06 ± 0.97	7.00 ± 1.20	.896
**VAS (mean ± SD)**			
Continuous	5.59 ± 2.27	5.13 ± 1.89	.515
Paroxysmal	8.29 ± 2.08	8.00 ± 2.07	.745
**MPQ (mean ± SD)**			
No. of descriptors	19.53 ± 1.28	19.75 ± 0.71	.740
Pain index	42.82 ± 11.92	41.38 ± 10.68	.773
**STAI (mean ± SD)**			
Trait	48.88 ± 8.28	50.88 ± 7.30	.866
State	44.71 ± 5.54	42.63 ± 4.03	.354
SF-36 (mean ± SD)	335.30 ± 154.87	320.25 ± 181.60	.332

Clinical and sociodemographic characteristics at baseline did not differ between the groups.BDI Beck Depression Inventory, DN4 DN4 questionnaire, VAS Visual Analogic Scale, MPQ McGill Pain Questionnaire, STAI State-Trait Anxiety Inventory, SF-36 SF-36 Quality of Life Questionnaire, MRC Medical Research Council.

No differences were found between the subgroups of sham stimulation (rTMS and tDCS) (*p* > 0.05), and therefore we combined them to form a single sham-stimulation group. In this sense, 3 groups were considered in the analysis of the results: active rTMS, active tDCS and sham stimulation.

### Primary outcome

#### Continuous pain

The comparison of changes in continuous pain intensity between rTMS, tDCS and sham stimulation showed a significant group (F = 4.94; *p* = 0.011; n_p_^2^ = 0.17) and time effect (F = 27.63; *p* < 0.001; n_p_^2^ = 0.37), with significant interaction between group and time (F = 8.41; *p* < 0.001; n_p_^2^ = 0.26). Post hoc Bonferroni tests indicated that rTMS and tDCS stimulation significantly decreased normalized scores of pain intensity compared with sham (*p* = 0.016 and *p* = 0.047, respectively). In addition, there was a significant improvement in pain relief at T1 and T2 for rTMS (*p* < .001 and *p* = 0.033, respectively) and tDCS (*p* < .001 and *p* = 0.005, respectively), when compared with T0.

More specifically, rTMS and tDCS were able to induce significantly stronger analgesic effects than sham stimulation after the fifth stimulation session (rTMS versus sham: t = 4.83; *p* < 0.001; d = 1.68; tDCS versus sham: t = 3.79; *p* < 0.001; d = 1.32). However, no differences between both active techniques were found (t = 0.45; *p* = 0.65; d = 0.15).

**Figure 3:**
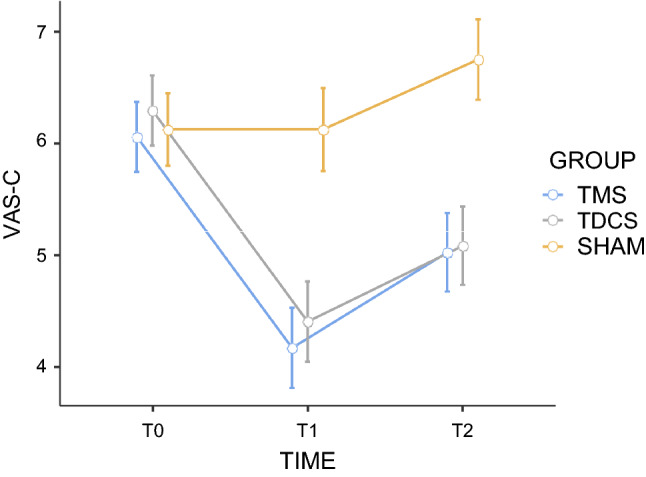
Effects of active repetitive transcranial magnetic stimulation (rTMS), active transcranial direct-current stimulation (tDCS), and sham stimulation on average continuous pain intensity (VAS). The scores were obtained before the first stimulation session (T0), after the 5th consecutive stimulation session (T1) and 30 days after (T2). For the sake of simplicity, and as the effects of sham rTMS and sham tDCS were remarkably similar regardless of the order in which they were conducted, we present the mean values grouped into a single sham-stimulation group. Error bars indicate standard error of the mean. VAS Visual Analogic Scale.

Paired comparisons after the 30-day interval (T2) showed that rTMS (t = 4.79; *p* < 0.001; d = 1.67) and tDCS (t = 4.48; *p* < 0.001; d = 1.56) were more effective than the sham stimulation, but no differences between both active techniques were found (*p* = 0.92) (Fig. 3).

#### Paroxysmal pain

**Figure 4:**
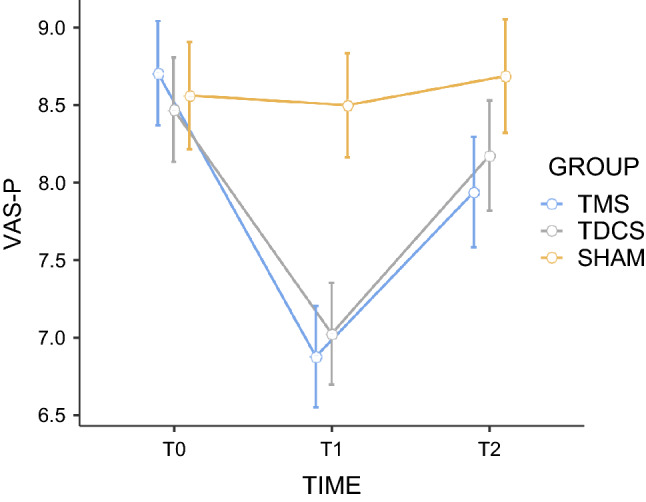
Effects of active repetitive transcranial magnetic stimulation (rTMS), active transcranial direct-current stimulation (tDCS), and sham stimulation on average paroxysmal pain intensity (VAS). The scores were obtained before the first stimulation session (T0), after the 5th consecutive stimulation session (T1) and 30 days after (T2). For the sake of simplicity, and as the effects of sham rTMS and sham tDCS were remarkably similar regardless of the order in which they were conducted, we present the mean values grouped into a single sham-stimulation group. Error bars indicate standard error of the mean. VAS Visual Analogic Scale.

According to Figure 4, the comparison of changes in paroxysmal pain intensity between rTMS, tDCS and sham stimulation showed a time effect (F = 18.52; < .001; n_p_^2^ = 0.283), but not a group effect (F = 2.05; *p* = 0.14; n_p_^2^ = 0.08), with significant interaction between group and time (F = 4.13; *p* = 0.004; n_p_^2^ = 0.15). Post hoc Bonferroni tests showed significant differences on scores of pain intensity in T1 compared with T0 (*p* < 0.001). In addition, there was a significant improvement in pain relief at T1 for rTMS (*p* < .001) and tDCS (*p* = 0.009), when compared with T0.

More specifically, rTMS and tDCS were able to induce significantly stronger analgesic effects than sham stimulation after the fifth stimulation session (rTMS versus sham: t = 3.48; *p* = 0.002; d = 1.21); tDCS versus sham: t = 2.37; *p* = 0.024; d = 0.83). However, no differences between both active techniques were found (*p* = 0.34) (Fig. 4).

### Secondary outcomes

#### Multidimensional aspect of pain

The comparison of changes regarding the scores achieved in evaluating the multidimensional aspect of pain (MPQ) between rTMS, tDCS and sham stimulation showed a significant group (F = 4.79; 0.013; n_p_^2^ = 0.17) and time effect (F = 14.72.63; *p* < 0.001; n_p_^2^ = 0.24), with significant interaction between group and time (F = 4.51; *p* = 0.002; n_p_^2^ = 0.16). Post hoc Bonferroni tests indicated that rTMS and tDCS stimulation significantly decreased normalized scores of MPQ index compared with sham (*p* = 0.022 and *p* = 0.041, respectively). However, such effect of active stimulations (rTMS and tDCS) was only observed after the last session (T1) (*p* < .001 and *p* = 0.048, respectively).

**Figure 5:**
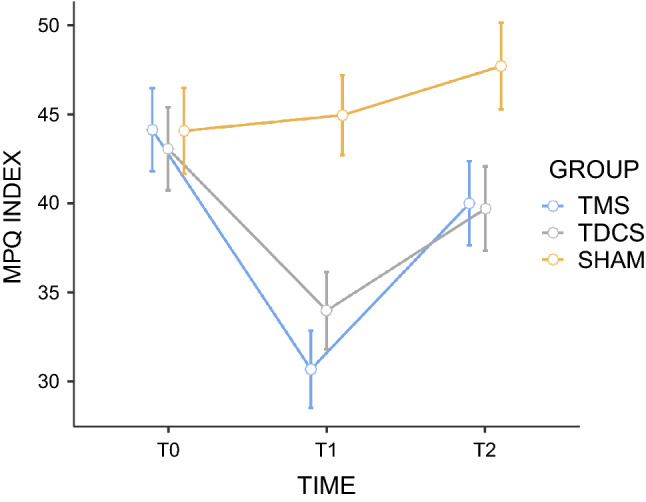
Effects of active repetitive transcranial magnetic stimulation (rTMS), active transcranial direct-current stimulation (tDCS), and sham stimulation on average multidimensional aspect of pain (MPQ). The scores were obtained before the first stimulation session (T0), after the 5th consecutive stimulation session (T1) and 30 days after (T2). For the sake of simplicity, and as the effects of sham rTMS and sham tDCS were remarkably similar regardless of the order in which they were conducted, we present the mean values grouped into a single sham-stimulation group. Error bars indicate standard error of the mean. MPQ McGill Pain Questionnaire.

More specifically, paired comparisons after the fifth session (T1) showed that rTMS (t = 3.53; *p* < 0.001; d = 1.23) and tDCS (t = 3.32; *p* = 0.002; d = 1.16) were more effective than the sham stimulation, but no differences between both active techniques were found (*p* = 0.27) (Fig. 5).

#### Anxiety state

The comparison of changes in the STAI-S measurements between rTMS, tDCS and sham stimulation showed a significant group (F = 5.53; *p* = 0.007; n_p_^2^ = 0.19) and time effect (F = 7.66; *p* < 0.001; n_p_^2^ = 0.14), with significant interaction between group and time (F = 8.67; *p* < 0.001; n_p_^2^ = 0.27). Post hoc Bonferroni tests indicated that TMS and tDCS stimulation significantly decreased normalized scores of anxiety state assessment compared with sham (*p* = 0.022 and *p* = 0.014, respectively). The achieved improvement levels in T1 were maintained after the 30-day interval (T2) for both active stimulations (*p* < 0.05).

**Figure 6:**
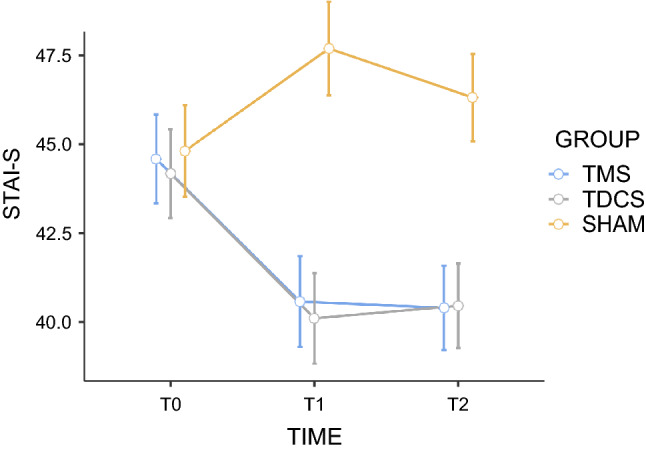
Effects of active repetitive transcranial magnetic stimulation (rTMS), active transcranial direct-current stimulation (tDCS), and sham stimulation on average anxiety state (STAI-S). The scores were obtained before the first stimulation session (T0), after the 5th consecutive stimulation session (T1) and 30 days after (T2). For the sake of simplicity, and as the effects of sham rTMS and sham tDCS were remarkably similar regardless of the order in which they were conducted, we present the mean values grouped into a single sham-stimulation group. Error bars indicate standard error of the mean. STAI-S State-Trait Anxiety Inventory (state subscale).

More specifically, paired comparisons showed that rTMS and tDCS were more effective than the sham stimulation after the last session (T1) (rTMS versus sham: t = 3.53; *p* = 0.001; d = 1.23; tDCS versus sham: t = 3.04; *p* = 0.005; d = 1.06) and after the 30-day interval (T2) (rTMS versus sham: t = 4.87; *p* < 0.001; d = 1.70; tDCS versus sham: t = 4.33; *p* < 0.001; d = 1.51), but no differences between active techniques in T1 and T2 were found (*p* = 0.88 and *p* = 0.68, respectively) (Fig. 6).

#### Quality of life

No difference was observed between the three types of stimulations regarding scores achieved in quality of life (SF-36) throughout the course of treatment (*p* > 0.05). ANOVA tests of each one of the eight SF-36 domain scales also revealed no significant improvement (*p* > 0.05).

#### Safety

The proportion of patients displaying side effects was low and similar between the groups (*p* > 0.05) (Table 2). No patients withdrew from the treatment because of such effects. Increase in pain scores was observed in some patients in all of the active and sham groups. Autonomic dysfunctions such as limb edema and worsening of Horner’s syndrome were reported in the active tDCS group.

**Table 2:** Side effects observed after stimulation sessions.

	Active rTMS (n = 12)	Sham rTMS (n = 8)	Active tDCS (n = 12)	Sham tDCS (n = 8)
Headache, n (%)	1 (8.33)		1 (8.33)	
Neck pain, n (%)			1 (8.33)	
Vertigo, n (%)	1 (8.33)			
**Increased pain, n (%)**				
*Continuous*	1 (8.33)	2 (25.00)	1 (8.33)	1 (12.50)
*Paroxysmal*		3 (37.50)	3 (33.33)	1 (25.00)
**Autonomic dysfunction, n (%)**				
*Limb edema*			1 (8.33)	
*Worsening of Horner’s syndrome*			1 (8.33)	

rTMS repetitive transcranial magnetic stimulation, tDCS transcranial direct-current stimulation.

## Discussion

In this preliminary study, we performed a double-blind, crossover and controlled investigation comparing rTMS versus tDCS effects in pain management. To our knowledge, this is the first known study to compare these two methods of non-invasive brain stimulation in NP located in the upper limb^12,14,18^. Overall, we observed that rTMS and tDCS applied in M1 are both effective in reducing continuous and paroxysmal NP in patients whose brachial plexus injury time ranged from 6 to 110.4 months. Such an analgesic effect also promoted improvement in MPQ, anxiety-state, but not in quality of life.

rTMS and tDCS aim to induce depolarization mechanisms in an attempt to reduce chronic pain, directly altering brain activity in an extensive neuronal network involved in pain processing^49^. Our results suggest similar modulation mechanisms are involved in reducing pain after brachial plexus injury, although the areas and pathways involved in each technique may be distinct, including the effect on the subtype of pain—continuous burning pain and paroxysmal shooting pain. The distinction between these NP patterns in this population is common in clinical practice and possibly involves different pathophysiological mechanisms^6,10,36,50^.

The finding that rTMS has an impact on pain deserves further investigation into the mechanisms of action. Intriguingly, the previous report indicates that epidural motor cortex stimulation (MCS) for BPA pain was ineffective for paroxysmal pain but moderately effective for continuous pain^50^. In BPA, paroxysmal pain is thought to originate from hyperactive neurons in the dorsal horn, whereas continuous pain is thought to originate from supraspinal structures, particularly the thalamus^6,50^. Nevertheless, neuronal hyperactivity has been also detected in thalamic nuclei, suggesting that supraspinal mechanisms contribute to paroxysmal shooting pain generation^51^. Furthermore, antinociceptive effects of rTMS, similar to MCS, can be mediated by the corticotalamic tract regardless of the functional integrity of the lemniscal system descending from the brainstem to the spinal cord (often compromised in BPA patients)^6,9,52^, explained not only the improvement in continuous pain but also in paroxysmal pain verified in rTMS active group.

Likewise, we obtained a significant result of tDCS in improving NP which affects the upper limb after brachial plexus injury. Regarding action mechanisms, up-regulation of motor cortex excitability by tDCS can induce remote indirect effects, not only in the thalamus, but, in particular, in the prefrontal and parietal areas^13,36,53,54^. However, few studies on NP^14,18^ proved to only be effective against individuals with lower-limb NP, such as that due to diabetic polyneuropathy or spinal cord injury when an anodal current of 2 mA was applied over the left M1 or contralaterally to the painful side^36,55,56^. It is probable that BPA mainly affects the central nervous system structures more than peripheral one^9^. Animal models of avulsion led to a more pronounced injury to the medial aspect of the Lissauer tract and the lateral dorsal column, with subsequent gliosis of the substantia gelatinosa that are closer to spinal cord lesions than to post-ganglionic injuries^9,57,58^. In addition, a positron emission tomography study has shown significantly increased metabolism after active tDCS in the medulla in patients with NP after spinal cord injury^56^. These mixed-mechanisms data may corroborate the pain relief achieved in our study.

Interestingly, rTMS improved MPQ. Based on previous evidence with MCS, multiple-session high-frequency rTMS over M1 is similarly capable to trigger a cascade of events of long time course involving perigenual cingulate and orbitofrontal areas, whose are considered critical for modulate the emotional appraisal of pain^12,16^. On the other hand, tDCS also shows benefits in MPQ scores. Possibly, anodal stimulation over M1 might modulate emotional and cognitive components of pain and normalize excessive attention to pain and pain-related information^56^.

The active treatment groups of both techniques showed better results compared to the placebo group in relation to anxiety state (STAI-S) and maintaining this improvement in the medium-term (30-day follow-up). Although anxiety associated with other neurological disorders^59^ such as BPI associated with chronic pain^11^ can negatively impact the quality of life, we do not obtain improvement in SF-36 scores. Indeed, the expected maintenance of physical disability throughout the course of treatment with non-invasive brain stimulation (NIBS) techniques^49^ probably contributed to not improving the quality of life. However, these results should be interpreted with caution and corroborated in future studies, including a longer time of stimulation and follow-up.

Although both techniques are non-invasive, tDCS has a lower cost, easier technical execution and more portability when compared to rTMS^20^, which has probably led to a growing increase in clinical research on tDCS over the last few years^14^, despite well-defined evidence for rTMS in NP^12,19^. In this sense, we obtained a favourable short-term result for both techniques in pain aspects (VAS and MPQ index) and anxiety state, as well as differences in the 30-day follow-up for continuous pain intensity and anxiety state, when compared to the sham stimulation; Perhaps, a more accurate and thorough knowledge of the correlation between the symptomatology and the pathophysiology of pain subtypes in brachial plexus injury might most certainly lead to further clinical progress and help in choosing the technique, since rTMS is not always available, which may provide greater use of tDCS in future clinical practice.

Our study has limitations which should be acknowledged. We did not employ neuroimaging and computational modelling techniques to control cortical changes or possible interference related to the disease neurophysiology. In order to minimize this bias, we controlled the eligibility criteria and randomized the groups and the order of sessions. Another limitation refers to the number of participants and the number of sessions performed group.

## Conclusions

Finally, the applicability of NIBS in this type of pain syndrome should be reproduced and better evaluated in future clinical trials with a larger number of participants and sessions in order to verify a long-lasting pain relief result in association with improvement in quality of life. Notwithstanding the above, our results highlight the potential use of tDCS for chronic pain management in traumatic brachial plexus injuries with little distinction from rTMS, which may promote the use of an easier and more available technique as part of an interdisciplinary approach in rehabilitation services for patients following upper limb deafferentation.

## Data Availability

The datasets generated during and analysed during the current study are not publicly available but are available from the corresponding author on reasonable request.
